# Science-based evidence on pathways and effects of human exposure to micro- and nanoplastics

**DOI:** 10.2478/aiht-2024-75-3807

**Published:** 2024-03-29

**Authors:** Buket Bakan, Nikolina Kalčec, Sijin Liu, Krunoslav Ilić, Yu Qi, Ivona Capjak, Lucija Božičević, Nikolina Peranić, Ivana Vinković Vrček

**Affiliations:** Institute for Medical Research and Occupational Health, Zagreb, Croatia; Atatürk University Faculty of Science, Department of Molecular Biology and Genetics, Erzurum, Turkey; Chinese Academy of Sciences Research Centre for Eco-Environmental Sciences, Beijing, China; Croatian Institute of Transfusion Medicine, Zagreb, Croatia

**Keywords:** adverse outcome, plastic particles, regulatory-relevant data, risk assessment, risk management, materijali plastike, nepovoljan ishod, procjena rizika, regulacijski značajni podatci, upravljanje rizikom

## Abstract

Human exposure to plastic particles has raised great concern among all relevant stakeholders involved in the protection of human health due to the contamination of the food chain, surface waters, and even drinking water as well as due to their persistence and bioaccumulation. Now more than ever, it is critical that we understand the biological fate of plastics and their interaction with different biological systems. Because of the ubiquity of plastic materials in the environment and their toxic potential, it is imperative to gain reliable, regulatory-relevant, science-based data on the effects of plastic micro- and nanoparticles (PMNPs) on human health in order to implement reliable risk assessment and management strategies in the circular economy of plastics. This review presents current knowledge of human-relevant PMNP exposure doses, pathways, and toxic effects. It addresses difficulties in properly assessing plastic exposure and current knowledge gaps and proposes steps that can be taken to underpin health risk perception, assessment, and mitigation through rigorous science-based evidence. Based on the existing scientific data on PMNP adverse health effects, this review brings recommendations on the development of PMNP-specific adverse outcome pathways (AOPs) following the AOP Users’ Handbook of the Organisation for Economic Cooperation and Development (OECD).

## Abstract (hr)

Izloženost ljudi česticama plastike izaziva veliku zabrinutost svih značajnih sudionika koji su uključeni u zaštitu ljudskoga zdravlja zbog onečišćenja prehrambenog lanca, površinskih voda, čak i pitke vode, kao i zbog njihove postojanosti i bioakumulacije. Stoga je ključno bolje razumijevanje biološke sudbine plastike i njezine interakcije s različitim biološkim sustavima. Zbog učestalosti materijala plastike u okolišu i njihove potencijalne toksičnosti, važno je prikupiti pouzdane, regulatorno značajne i znanstveno utemeljene podatke o učincima mikro- i nanoplastike na ljudsko zdravlje kako bi se pravilno primijenile pouzdane strategije procjene rizika i upravljanja rizicima u kružnoj ekonomiji lanca vrijednosti plastike. Ovaj pregledni rad predstavlja trenutačno znanje o dozama izloženosti mikro- i nanoplastici relevantnima za ljude, oblicima izloženosti, kao i o njezinim toksičnim učincima. Istaknute su poteškoće u pravilnoj procjeni problema izloženosti plastici i trenutačni nedostatci u znanju te su predloženi potencijalni koraci koji se mogu poduzeti kako bi se podržala percepcija, procjena i ublažavanje rizika za zdravlje strogim znanstveno utemeljenim dokazima. Na temelju postojećih znanstvenih podataka o štetnim učincima mikro- i nanoplastike na zdravlje, daju se preporuke za razvoj specifičnih načina nepoželjnih ishoda (eng. *adverse outcome pathway*, krat. AOP) za mikro- i nanoplastiku prema priručniku korisnika AOP-a Organizacije za ekonomsku suradnju i razvoj (izv. Organisation for Economic Co-operation and Development, krat. OECD).

Plastic pollution has long been one of the top environmental issues due to its abundance and persistence in the aquatic environment, which threatens to double over the next 20 years in a business-as-usual scenario (1), whether it slowly degrades to or is released into the environment as industrial plastic micro- and nanoparticles (PMNPs) (e.g., paints, adhesives, electronics, and cosmetics) (2,3,4,5,6).

Chronic exposure to plastic particles raises great concern for all life, as we interact with them without completely understanding how they affect our planet and much less our bodies through the food chain and surface or even drinking water (3,4,5,6,7,8,9,10). The smaller they are the more easily they penetrate blood, endothelial, intestinal, blood-brain, and blood-placental barriers to reach and accumulate in tissues and organs with different adverse outcomes. Their uptake by different body compartments also depends on their shape and surface chemistry (11).

Because of the ubiquity of plastic materials and their toxic potential, it is imperative to gather reliable, regulatory-relevant, and science-based evidence on how PMNPs affect human health to properly assess and manage risks in the context of circular economy. The main regulatory pathways to collect such data should follow the Integrated Approaches to Testing and Assessment (IATA) supported by the adverse outcome pathway (AOP) concept (12, 13). In 2019, the World Health Organization (WHO) reviewed the state of evidence on PMNPs in drinking water (14), while the European Commission adopted its strategy for plastics in a circular economy in 2018 (15). However, the main challenge is the lack of sensitive analytical methods to detect and quantify PMNPs in different biological and environmental matrices. It is impossible to assess PMNP exposure, toxicokinetics, and toxicodynamics without measuring the number and concentration of particles that reach and accumulate in biological fluids, cells, tissues, and organs. So far, dose-response data in humans are very scarce.

This review aims to synthesise current knowledge on PMNP exposure doses, pathways, and health effects in humans. We also highlight current knowledge gaps and the challenges in assessing plastic exposure and bring recommendations for the development of PMNP-specific AOPs based on the existing scientific evidence on PMNP adverse health effects.

## METHODOLOGY

The first step towards collecting current science-based evidence of PMNP effects on human health was a comprehensive literature search across the Web of Science Core Collection (WoSCC) database, using the search phrase “(micro OR nano) AND (plast*) AND (tox*) AND (“*in vivo*” OR human)”. The search was further refined by the additional phrases “(“mur*” OR “mouse” OR “rat”)”, to obtain results published on *in vivo* rodent animal models. The database was accessed on 28 March 2023, and the search identified a total of 343 papers. They were screened, and non-relevant papers excluded to finally get 43 relevant papers reporting qualitative data on PMNP health effects and quantitative data on PMNP uptake, of which 26 were review articles. Due to the scarcity of human-related experimental data, we decided to include and analyse data from *in vivo* and *in vitro* studies to fully appreciate the scope of health issues that could be associated with plastic materials and PMNPs. Therefore, our analysis includes all data relating to the magnitude of exposure (i.e., number of particles taken up by cells, tissues, or organisms) and PMNP effects on cell or tissue metabolism.

**Figure 1:**
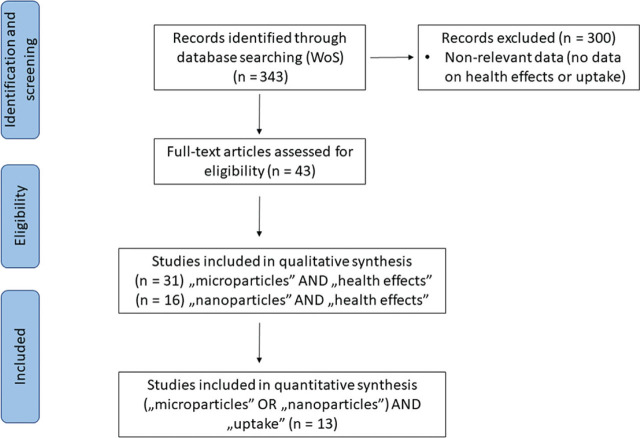
Flow chart of literature search in the Web of Science Core Collection (WoSCC) database

In addition to the literature search in the WoSCC database, we reviewed information in the AOP-Wiki (16) to identify AOPs that may be associated with the health effects reported for PMNPs. This work was based on comparing adverse effects reported in the 43 WoSCC papers (Figure 1) with molecular initiating events (MIEs) or key events (KEs) described in the AOP-Wiki for different AOPs. The value of the AOP framework lies in the fact that AOPs are not specific for particular stressors but can define downstream PMNP effects if proper MIEs or KEs are identified. AOPs can facilitate risk assessment and management by placing mechanistic knowledge in hazard characterisation in a specific regulatory context (17, 18).

Moreover, identifying AOPs relevant for PMNPs can further inform the choice of the most suitable assays for assessing specific adverse outcomes (19). As already demonstrated by several IATA OECD case studies describing AOPs for non-genotoxic carcinogens, skin sensitisation, chemical-induced liver steatosis, and neural development (17), implementation of the AOP framework may improve non-animal *in vitro* testing for future hazard characterisation of PMNPs.

## RESULTS AND DISCUSSION

### Routes of exposure and estimated human uptake of PMNPs

**Figure 2:**
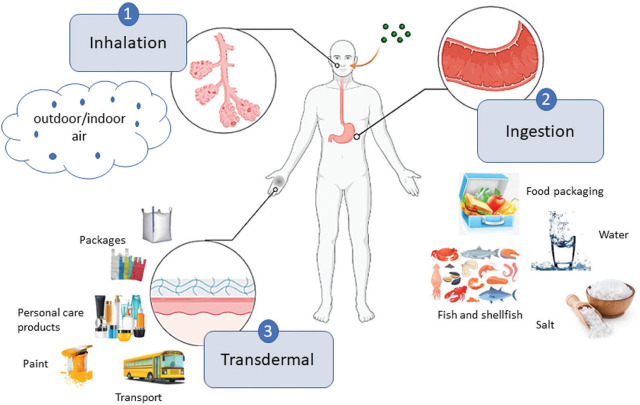
Human exposure routes to plastic micro- and nanoparticles in humans (created with BioRender. com)

Data on routes of human exposure (Figure 2) show that inhalation and ingestion are much more relevant for PMNP uptake than the transdermal route (20, 21). There are many sources of inhalation exposure, depending on the type of contamination of indoor and outdoor air. Inhaled PMNPs can translocate into the lung tissue or enter the digestive system via mucociliary clearance (7, 22, 23). Oral uptake is mostly owed to contamination of bottled drinks or different food items including table salt (24, 25). PMNPs can also contaminate food during food processing and packaging.

For all exposure routes, the mechanism of uptake depends on PMNP size, shape, solubility, and surface characteristics as well as on biological factors, such as the site of particle deposition (7, 26). Depending on the size, PMNPs can spread through passive diffusion, paracellular transfer, or active cellular uptake (7), which also depends on the cell type, e.g., Peyer’s patches in the intestine or alveolar macrophages in the lung (7, 27). Even though dermal contact accounts for a relatively small fraction of PMNP uptake, some studies suggest that PMNPs can be absorbed through hair follicles and sweat ducts, which could lead to systemic exposure (28,29,30).

Estimations of PMNP intake by humans significantly vary between studies (Table 1) and depend on the type of PMNP and exposure route(s) considered by a particular study. For example, individual dietary PMNP intake in the USA is estimated to 39,000–52,000 particles per year, while the total intake increases to 74,000–121,000 particles per year if we include the inhalation route (31). It is important to note that all estimations presented in Table 1 are based on limited data and may not be representative of a specific population or geographic region. Furthermore, reliable, accurate, and precise methods for identification, characterisation, and quantification of PMNPs in complex matrices like environmental and biological samples are still scarce (8, 14, 15, 20, 32). This problem is especially frustrating for the development and implementation of particle dosimetry in PMNP risk assessment and management whose aim is to quantify the number of plastic particles in a particular analytical sample.

**Table 1:** Overview of human-relevant exposure pathways for plastic micro- and nanoparticles with numerical data for levels of intake where available

**Biological models**	**Exposure data**	**Reference**
Ecotoxicity, human cells, humans	Ingestion route: 11,000 particles from shellfish, 4000 particles from drinking water, and 7–1000 particles from edible sea salt per person per year	(36)
Ecotoxicity, human cells, humans	Ingestion route. Sources: seafood, tea bags, honey, sugar, beverage drinks, commercial salts, milk, beer, tap and bottled drinking water	(37)
Ecotoxicity, humans	Ingestion sources: drinking water, food containing plastic particles or weathering from plastic containers, salts and honey, and beer	(5)
Ecotoxicity, human cells and exposure	Ingestion through the food chain	(27, 31, 38,39,40,41,42,43,44,45)
Human exposure	Ingestion route: ≤30 particles/day from tap water and beverages, 37–100 particles/year from sea salt; in total ≤250 pg/kg body weight per day for an adult from tap water, beverages, and sea salt	(11)
Rodent model	5-day oral exposure to 60 nm polystyrene particles: 10 % of the dose found in the gastrointestinal tract	(46)
*In vitro* models	Exposure experiments with red blood cells, peripheral blood mononuclear cells, and mast cells	(47)
*In vitro* models	Exposure experiments with intestinal epithelial cell lines, LS174T, HT-29, and CaCo-2	(22, 48)
*In silico* models	/	(49)
Invertebrates and vertebrates	Exposure through the food chain	(50)
*In vitro* models	Exposure experiments with epithelial HeLa and cerebral T98G cells	(51)
*In vivo* model organisms	Ingestion route: 12,000–204,000 particles per person per year via plastic-contaminated seafood (fish and shellfish), beer, table salt, sugar, and honey	(52)
*In vitro* model of the whole digestive system	Annual ingestion: 123,000 particles for adults (714 mg/day), 81,000 particles for children (449 mg/day).	(53)
*In vitro* models, animal models, plants	Ingestion via the food chain	(54, 55)
Human exposure	Annual ingestion: 11,000 particles from shellfish for European top consumer. Ingestion sources: 50.97 particles/L of beer, 24.53 particles/L of soft drinks, 5.79 particles/L of energy drinks, 5.26 particles/L of cold tea, 3 to 11 particles/L of milk; the highest mean concentrations of particles from drink consumption in the US (9.24–11.8 particles/L) and the lowest in Germany (0.91–1.29 particles)	(56)
*In vivo* animal models, mammals	The primary route of exposure: ingestion of food and water contaminated with PMNP; annual consumption of 39,000 and 52,000 particles per person in the US	(57)
*In vivo*	Ingestion of PMNP does not provide a significant contribution to the transfer of absorbed chemicals from the water to the biota via the gut	(10)
*In vitro*, *in vivo*	The oral bioavailability of 50 nm-sized polystyrene particles differs between 0.2 and 2 % in rodents (*in vivo*) and humans (*in vitro*); a relationship between the particle’s composition, size, and uptake has not yet been established	(58)
*In vivo* ecotoxicity (fish)	Estimated exposure from the consumption of *Sufflamen fraenatus*, *Heniochus acuminatus*, *Pseudotriacanthus, Leiognathus brevirostris,* and *Atropus atropus* for adults: 456, 310, 213, 156 and 121 particles per week, respectively and children: 68, 45, 32, 25 and 19 of particles per week, respectively	(59)
*In vivo*, human	Annual ingestion via shellfish: 11,000 particles for the European population. It is also reported that a regular consumer of sea salt ingests approximately 37 synthetic fibres daily	(60)
*In vitro*, *in vivo*, human	Estimated exposure for adults: 258 to 312 particles daily	(26)
*In vitro* intestinal barrier	Uptake of ≤0.144 % of polystyrene microparticles across the Caco-2 monolayer	(2, 61)
*In vitro*	Absorption of more than 70 % of nanoparticles with a significant reduction of rate to 30–50 % for microparticles	(62)

Due to the resistance of plastic materials to degradation, bioaccumulation should be considered a serious health issue, as inhaled and ingested plastic particles may remain in different tissues for long and cause chronic health effects (28,29,30, 33). Deng et al. (34) reported that plastic microparticles in the liver, kidney, and gut tissue accumulate in a range of 0.07–0.41 mg of plastic material per gram of tissue. Another animal study (23) reported very low accumulation in the intestinal cell layer and no plastic materials in the liver, spleen, or kidney. Unfortunately, most studies report difficulties in properly assessing the toxicokinetic/toxicodynamic profiles of PMNPs (35).

### Health effects of PMNPs

According to the reviewed scientific literature (Figure 1), PMNPs may cause various detrimental effects on cell viability, inflammatory response, lipid metabolism, oxidative status, intestinal microbiota, ion transport, signalling pathways, DNA integrity, hepatic function, and more. Table 2 lists the reported PMNP effects on human and animal cells and tissues. The sheer number of different adverse health effects following exposure to PMNPs raises concern about the ever-increasing plastic pollution.

**Table 2:** Science-based evidence of adverse health effects of plastic micro and nanoparticles (PMNP)

**Models**	**Type of plastics**	**PMNP biological effects**	**Reference**
Ecotoxicity, human cells, human exposure	PE-, PS, PVC-, PET- and PLGAMPs; PS-NPs	Reactive oxygen species (ROS) production (63, 64), activation of antioxidant enzymes (50), increase in glutathione S-transferase (GST) activity (65) and mitogen-activated proteins kinase signalling pathways (66), a decline in lipid digestion and inhibition of digestive enzymatic activities (67), impact on the cell morphology and cell proliferation of immune cells (68, 69)	(36)
Ecotoxicity, human cells, human exposure	PE- and PS-MPs	DNA damage via oxidative stress, distortion of cellular proteins involved in cell division, an aberration in signalling responses, down-regulation of transcriptional genes related to apoptotic expressions (70), increased DNA fragmentation in liver tissue, altered activity of antioxidant enzymes and increased lipid peroxidation (71, 72)	(37)
Ecotoxicity, human cells	PS-MPs; PS-NPs	Disturbance in lipid metabolism, oxidative stress and neurotoxicity (34), aggregation of serum proteins (73)	(5)
Ecotoxicity, human blood cells	PS-NPs	Conformational changes in blood proteins, cytotoxic and genotoxic effects in lymphocytes and erythrocytes	(74)
Ecotoxicity	MPs of few microns or less	Adsorption of proteins and local inflammation in the gastrointestinal system (75)	(27)
Ecotoxicity, human exposure	Nylon fibres of a respirable size; PE-MPs	Persistent inflammation (7)	(41)
Ecotoxicity, animal models, human exposure	PS- and PEMPs; PS-NPs	Decrease in hepatic triglyceride and total cholesterol levels, decrease in gene expression related to lipogenesis and triglyceride synthesis in liver, reduction of intestinal mucus secretion (76), metabolic disorders due to alteration of intestinal microbiota (77), induction of IL-6 and IL-8 expression in gastric adenocarcinoma cells (68), induction of oxidative stress inT98G cells (63), increased AChE activity in the liver (34)	(42)
*In vivo* animal models	PS-MPs	Absence of histologically detectable lesions and no inflammatory responses in mice (23)	(43)
*In vitro* CaCo-2 & THP-1 / T98G & HeLa human cells	PS- and PE-MPs	No cytotoxicity (23, 63)	(39)
*In vitro* model	PS-NPs	Inflammation, oxidative stress, lysosomal dysfunction and apoptosis in cultured cells	(11)
*In vitro* (mouse hepatocytes)	PS-NPs	Oxidative stress and DNA damage (78)	(39)
Invertebrates and vertebrates	PS-, PE-, HDPE-MPs	Decreased mucus secretion and mucus secretion-related gene expression (79), down-regulation of genes related to ion transport (76), modified serum levels of IL1α and granulocyte colony-stimulating factor G-CSF, decreased regulatory T cell count and increased the proportion of Th17 cells in splenocytes (80), blood neutrophil counts and IgA levels elevated in dams, and spleen lymphocytes were altered in both dams and offspring (81)	(46)
*In vitro* [red blood cells, peripheral blood mononuclear cells (PBMCs), mast cells]	PS-MPs	No cytotoxic effects on PBMCs and mast cells, haemolysis of erythrocytes, increased IL-6 production	(47)
*In vitro* (intestinal epithelial cell lines, LS174T, HT-29, and Caco-2)	PS-NPs	Reduced cell viability	(22)
Rodents	PS-MPs	Accumulation in the liver, kidney, and gut; energy and lipid metabolism disorders, liver inflammation (34), decreased intestinal mucus, changes in intestinal biota (77, 79)	(82)
*In vitro* (epithelial HeLa and cerebral T98G cells) and rodents	PS-MPs	Cells: binding of blood plasma coagulation factors VII and IX leading to a decrease in thrombin generation Mice: accumulation in the liver, kidney and gut with evidence of oxidative stress, energy balance disturbance, and neurotoxicity (83, 84)	(51)
*In vivo* model organisms	PS- and PE-MPs	Blood clots (85), blood cell cytotoxicity (86), oxidative stress (34, 87)	(50)
*In vitro* (CaCo-2 cells)	PET-NPs	No effect on cell viability and membrane integrity, no significant change in apoptosis and necrosis	(48)
*In vivo*, animal models	PS-MPs	Altered hepatic lipid metabolism, decrease in body weight, liver weight, decreased serum triglycerides and total cholesterol, mucus secretion, changes in gut microbiota, impairment of bile acid metabolism (77, 79)	(2)
*In vivo*, animal models	PS-MPs	Mechanical injury, false satiation, low growth rate, increased immune response, energy depletion, blocked enzyme production, decreased fecundity, oxidative stress, morbidity and mortality (88, 89)	(55)
*In vivo*, animal models	PS-MPs; PS-NPs	Inflammatory response, reduced intestinal mucus secretion, damage to the intestinal barrier function leading to an increase in the permeability of the gut mucosa, trigger an imbalance of gut microbiota, and alter metabolism, such as lipogenesis, triglyceride synthesis (77, 79), induction of pro-inflammatory responses (pro-inflammatory cytokines IL6, IL8 and IL1β), and inhibition of cell viability (68)	(54)
*In vivo*	LDPE-MPs	No toxic effects	(90)
*In vitro* (CaCo-2 cells)	PLA particles	No cytotoxicity and no altered cell viability	(9)
*In vivo*, human	Plastic fibres; PS-MPs	Bioaccumulation of synthetic microfibres in the gastro-intestinal tract and lungs of humans (91), inflammation, genotoxicity, oxidative stress, and apoptosis in the human body (92)	(60)
*In vitro*, *in vivo*	PS-MPs	*In vitro*: no effects on the phosphorylation of STAT-1 and STAT-6, no effect on the expression of CXCL10 and CCL22, CD209 and CD206 genes. Mice – no statistically significant effects on body and organ weights, no effect on tissue morphology	(23)
*In vitro* (CaCo-2 cells)	PS-MPs; PS-NPs	Increase in intracellular ROS levels, mitochondrial depolarisation, and increased cytotoxicity	(93)
Ecotoxicity	PS-NPs	Up-regulation of cytokines involved in gastric pathologies (68), disruption of iron transport (94), induction of apoptosis (22), endoplasmic reticulum stress (95) and oxidative stress (96)	(97)
*In vitro*	Molecularly imprinted polymers; PS-NPs	No significant toxicity	(98, 99)

HDPE – high-density polyethylene; LDPE – low-density polyethylene; MPs – microplastics; NPs – nanoplastics; PE – polyethylene; PET – polyethylene terephthalate; PLA – polylactic acid; PLGA – poly(lactic-co-glycolic acid); PS – polystyrene; PVC – polyvinyl chloride

### Linking PMNP-induced health effects to existing AOPs for chemicals

The AOP framework (13) enables collection and logical organisation of experimental data from different sources for efficient identification of essential biological events affected by exposure of living organisms to chemical stressors. Here we used the AOP framework to identify molecular initiating events (MIEs) and key events (KEs) associated with biological effects of PMNPs given in Table 2. The main aim was to generate hypothesis-based AOPs relevant to PMNPs by linking PMNP-induced toxicity endpoints reported so far with existing AOPs in the AOP-Wiki (16). However, it is important to note that many of these AOPs are under development.

Our analysis shows that PMNPs reported to induce biological effects have already been described either as MIEs or as KEs in 170 different AOPs (Table 3), which is an alarming finding that urges for more focused risk assessment of plastic across all steps in its value chain.

**Table 3:** AOPs in AOP-Wiki related to the observed PMNP effects found in the literature search (see Figure 1)[Reported biological effects of PMNP are denoted as molecular initiating events (MIE) or key events (KE) for each AOP. AOPs marked in the last column with an asterisk are “under development”]

**Reported biological effect of PMNP**	**Adverse outcome (AO)**	**Total number of AOPs**	**Event type in identified AOPs**
Increased reactive oxygen species (ROS); oxidative stress induction	Decreased population growth rate/Decreased population size	**8**	**MIE** in AOP 444*; **KE** in AOP 386*, AOP 387*, AOP 396*, AOP 299*, AOP 311*, AOP 325*, AOP 326*
	Lung fibrosis/Lung cancer/Decreased lung function/Dysfunction of the respiratory system	**7**	**MIE** in AOP 481*; **KE** in AOP 382*, AOP 319*, AOP 303*, AOP 416*, AOP 451*, AOP 418*
	Liver fibrosis/Cholestasis/Steatohepatitis/Liver injury	**5**	**KE** in AOP 383*, AOP 27*, AOP 213 (open for adoption), AOP 273*, AOP 494*
	Breast cancer	**2**	**MIE** in AOP 294*; **KE** in AOP 293*
	Increased mortality	**7**	**MIE** in AOP 327*, AOP 328*, AOP 329*, AOP 330*, AOP 186*; **KE** in AOP 377*, AOP 413 (open for citation & comment)
	Reproductive failure/Decreased fertility/Decreased reproductive success/Impaired fertility	**4**	**KE** in AOP 207*, AOP 476*, AOP 473*, AOP 492*
	Treatment-resistant gastric cancer	**1**	**MIE** in AOP 298 (under review)
	Decreased cognitive function	**1**	**MIE** in AOP 488*
	Inflammatory events in light-exposed tissues	**1**	**MIE** in AOP 282 (under review)
	Insulin resistance	**2**	**KE** in AOP 457*, AOP 497*
	Chronic kidney disease	**1**	**KE** in AOP 384*
	Apoptotic cell death	**1**	**MIE** in AOP 423*
	Increased oxidative damage	**1**	**KE** in AOP 26 (open for adoption)
	Increased mesotheliomas	**1**	**KE** in AOP 409*
	Acute myeloid leukaemia	**1**	**KE** in AOP 432*
DNA damage/DNA strand breaks	Decreased population growth rate/Decreased population size/Decreased population trajectory	**6**	**KE** in AOP 388*, AOP 444*, AOP 216*, AOP 238*, AOP 396*, AOP 435*
	Lung cancer	**5**	**KE** in AOP 303*, AOP 451*, AOP 416*, AOP 417*, AOP 272 (Approved)
	Breast cancer	**4**	**MIE** in AOP 443*, AOP 293*, AOP 294* **KE** in AOP 200 (open for adoption)
	Decreased fertility/Reduced sperm count	**2**	**KE** in AOP 476*, AOP 322*
	Increased mesotheliomas	**1**	**KE** in AOP 409*
	Acute myeloid leukaemia	**1**	**KE** in AOP 432*
	Microcephaly	**1**	**KE** in AOP 441*
	Apoptotic cell death	**1**	**KE** in AOP 423*
	Increased chromosomal aberrations	**1**	KE in AOP 296 (endorsed)
	Cataracts	**1**	**KE** in AOP 478 (open for citation & comment)
	Learning and memory impairment	**1**	**KE** in AOP 483 (open for citations & comments)
	Vascular remodelling	**1**	**KE** in AOP 470 (open for citations & comments)
Increased lipid peroxidation	Increased mortality	**2**	**KE** in AOP 413 (open for citation & comment), AOP 329*
	Impaired fertility	**1**	**KE** in AOP 492*
Apoptosis	Decreased population growth	**3**	**KE** in AOP 340*, AOP 341*, AOP 444*
	Decreased fertility/Reproductive failure/Decreased reproduction	**4**	**KE** in AOP 70*, AOP 71*, AOP 207*, AOP 476*
	Orofacial cleating	**2**	**KE** in AOP 460*, AOP 491*
	Breast cancer	**2**	**KE** in AOP 200 (open for adoption), AOP 439*
	Decreased lung function/Chronic obstructive pulmonary disease	**2**	**KE** in AOP 419*, AOP 452*
	Prostate cancer	**1**	**KE** in AOP 495*
	Apoptosis/Necrosis	**1**	**AO** in AOP 205 (open for comment)
	Testicular atrophy	**1**	**KE** in AOP 212 (endorsed)
	Liver injury	**1**	**KE** in AOP 285*
	Thyroid hormone interference	**1**	**KE** in AOP 393*
	Neurodegeneration	**1**	**KE** in AOP 260*
	Kidney failure	**1**	**KE** in AOP 447*
Lysosomal disruption	Kidney toxicity	**1**	**KE** in AOP 257*
	Liver fibrosis	**1**	**KE** in AOP 144 (under review)
Decreased locomotor activity	Decreased population growth rate	**1**	**KE** in AOP 218 (not under active development)
Decrease in body weight	Decreased body weight	**1**	**AO** in AOP 6 (endorsed)
Glutathione depletion	Impaired fertility	**1**	**KE** in AOP 492 *
Inflammation/Neuroinflammation	Learning and memory impairment	**4**	**KE** in AOP 12 (endorsed), AOP 17 (endorsed), AOP 48 (endorsed), AOP 490*
	Parkinsonian motor deficits	**2**	**KE** in AOP 3 (endorsed), AOP 464*
	Lung fibrosis/Bronchiolitis obliterans/Chronic obstructive pulmonary disease	**3**	**KE** in AOP 206*, AOP 280*, AOP 452*
	Cholestasis/Immune-mediated hepatitis/Increased liver steatosis	**2**	**KE** in AOP 413*, AOP 362* **MIE** in AOP 62*
	Increase, papillomas/carcinomas	**2**	**KE** in AOP 114*, AOP 115*
	Psoriatic skin disease	**1**	**KE** in AOP 313*
	Increased mortality	**1**	**KE** in AOP 377*
	Hyperinflammation	**1**	**AO** in AOP 392*
	Memory Loss	**1**	**KE** in AOP 429*
	Kidney failure	**1**	**KE** in AOP 447*
	Metabolically unhealthy obesity	**1**	**KE** in AOP 493*
	Increased mesotheliomas	**1**	**KE** in AOP 171*
	Breast cancer	**1**	**KE** in AOP 439*
Pulmonary inflammation	Chronic obstructive pulmonary disease	**1**	**KE** in AOP 452*
	Respiratory dysfunction	**1**	**KE** in AOP 481*
	Increased mesotheliomas	**1**	**KE** in AOP 409*
	Increased mortality	**1**	**KE** in AOP 377*
	Hypersensitivity response	**1**	**KE** in AOP 39*
	Atherosclerosis	**1**	**KE** in AOP 237*
Increased neutrophil activation	Increased thrombo-inflammation	**1**	**KE** in AOP 412*
Mitochondrial dysfunction	Death/Failure, Colony	**6**	**KE** in AOP 77 (open for comment), AOP 78 (open for comment), AOP 79 (open for comment), AOP 80 (open for comment), AOP 87 (open for comment), AOP 178*
	Decreased population growth rate	**3**	**MIE** in AOP 326*, AOP 325*, AOP 324*
	Heart failure/Increased mortality	**3**	**KE** in AOP 479*, AOP 480*, AOP 377*
	Kidney failure/Kidney toxicity	**2**	**KE** in AOP 447*, AOP 437*
	Liver fibrosis/Liver injury	**2**	**KE** in AOP 144 (under review), AOP 273*
	Learning and memory impairment	**1**	**KE** in AOP 48 (endorsed)
	Parkinsonian motor deficits	**1**	**KE** in AOP 3 (endorsed)
	Breast cancer	**1**	**KE** in AOP 200 (open for adoption)
	Apoptotic cell death	**1**	**KE** in AOP 423*
Impaired IL-1R signalling	T-cell-dependent antibody response impairment	**1**	**MIE** in AOP 277 (under review)
Energy reserves depletion/Decreased fatty acid beta-oxidation	Decreased population growth rate	**1**	**KE** in AOP 97*
	Decreased body weight	**1**	**KE** in AOP 6 (endorsed)
Blocked enzyme production (inhibition of aromatase/calcineurin activity)	T-cell-dependent antibody response impairment	**1**	**KE** in AOP 154 (endorsed)
	Metastasis, Breast Cancer	**1**	**KE** in AOP 443*
	Decreased fertility	**1**	**MIE** in AOP 153*
Decreased fecundity/fertility	Decreased fecundity/Decreased population growth rate/Decreased fertility	**13**	**AO** in AOP 73*, AOP 126*, AOP 345*, AOP 476*, AOP 64*, AOP 66*, AOP 67*, AOP 68*, AOP 70*, AOP 71*, AOP 74*, AOP 153* **KE** in AOP 444*
Oxidation of iron in haemoglobin	Cyanosis	**1**	**MIE** in AOP 31*
Endoplasmic reticulum stress	Non-alcoholic fatty liver disease/Tumorigenesis	**1**	**KE** in AOP 454*
	Parkinsonian motor deficits	**1**	**KE** in AOP 464*
Decreased cholesterol/altered cholesterol metabolism	Impaired fertility	**2**	**KE** in AOP 51*, AOP 18 (under review)
	Decreased sperm quantity and/or quality in the adult testis	**1**	**MIE/KE** in AOP 69*
	Decreased cognitive function	**1**	**KE** in AOP 487*
Altered lipid metabolism	Steatohepatitis	**1**	**KE** in AOP 401*
	Pancreatic acinar tumours	**1**	**KE** in AOP 166*
Metabolic syndrome/stress	Metabolic syndrome/Insulin resistance	**1**	**AO** in AOP 497*
	Death/Failure, Colony	**1**	**KE** in AOP 81 (open for comment)
Increased blood CCK level (satiety)	Pancreatic acinar cell tumours	**1**	**KE** in AOP 316*
Cell injury	Learning and memory impairment	**5**	**KE** in AOP 48 (endorsed), AOP 13 (endorsed), AOP 12 (endorsed), AOP 17 (endorsed), AOP 490*
	Liver fibrosis/Liver injury/	**5**	**KE** in AOP 38 (endorsed), AOP 144 (under review), AOP 278*, AOP 273*, AOP 494*
	Decreased growth	**3**	**KE** in AOP 265*, AOP 264*, AOP 266*
	Heart failure/Increased mortality	**2**	**KE** in AOP 479*, AOP 377*
	Neurodegeneration	**1**	**KE** in AOP 281*
Inhibition of digestive enzymatic activities (trypsin inhibition)	Pancreatic acinar cell tumours	**1**	**MIE** in AOP 316*
Alteration in intestinal microbiota	Gut dysbiosis	**1**	**KE** in AOP 428*
Decrease in gene expression related to liver	Increased hepatocellular adenomas and carcinomas	**1**	**KE** in AOP 107 (under review)
Covalent protein binding	Sensitisation of skin	**1**	**MIE** in AOP 40 (endorsed)
	Increased allergic respiratory hypersensitivity response	**1**	**MIE** in AOP 39*
Meiotic spindle disorganisation	Increased aneuploid offspring	**1**	**KE** in AOP 106 (EAGMST under review)
Increased mortality	Increased mortality	**23**	**AO** in AOP 16*, AOP 96*, AOP 104*, AOP 113*, AOP 160*, AOP 161*, AOP 138*, AOP 177*, AOP 186*, AOP 312*, AOP 155 (endorsed), AOP 156 (endorsed), AOP 157 (endorsed), AOP 158 (endorsed), AOP 159 (endorsed), AOP 363 (under review), AOP 377*, AOP 364 *, AOP 365 *, AOP 399 (open for citation & comment), AOP 413 (open for citation & comment), AOP 410 (open for citation & comment), AOP 450*
Decreased population growth rate	Decreased population growth rate	**54**	**AO** in AOP 23 (endorsed), AOP 25 (endorsed), AOP 29*, AOP 30 (under review), AOP 100*, AOP 122(under development), AOP 123*, AOP 155 (endorsed), AOP 156 (endorsed), AOP 157 (endorsed), AOP 158 (endorsed), AOP 159 (endorsed), AOP 101*, AOP 102*, AOP 63*, AOP 103*, AOP 310*, AOP 16*, AOP 312*, AOP 334*, AOP 336*, AOP 337*, AOP 338*, AOP 339*, AOP 340*, AOP 341*, AOP 297*, AOP 346*, AOP 326*, AOP 325*, AOP 324*, AOP 363 (under review), AOP 348*, AOP 376 (open for citation & comment), AOP 386*, AOP 387*, AOP 388*, AOP 389*, AOP 364*, AOP 365*, AOP 399 (open for citation & comment), AOP 410 (open for citation & comment), AOP 216 *, AOP 238*, AOP 299*, AOP 311*, AOP 444*, AOP 138*, AOP 177*, AOP 97*, AOP 203*, AOP 218 (not under active development), AOP 219 (not under active development), AOP 323 (open for citation & comment)

AOP – adverse outcome pathway; KE – key event; MIE – molecular initiating event

## CONCLUSIONS

The reviewed literature clearly evidences adverse health effects of PMNPs, even though they are not fully understood in humans. Many studies demonstrate that PMNPs can accumulate and cause inflammation in various tissues and organs or disrupt cellular processes.

It is important to consider all routes to determine aggregate exposure and include all routes in future studies of PMNP health effects. Risk assessment should also include interactions between PMNPs and other environmental pollutants that may act as a Trojan horse introducing hazardous substances into the body.

Research and action are needed to address all these issues and minimise the risks associated with the ever-increasing use of plastic materials. Further studies are needed to fully understand the mechanisms by which PMNPs affect human health and the extent of their toxicity at various exposure levels. Long-term studies are also needed to determine their chronic effects in order to develop effective risk assessment and management strategies and inform policy decisions aimed at minimising exposure to these particles and protecting human health.
